# IGF2BP2 promotes head and neck squamous carcinoma cell proliferation and growth via the miR-98-5p/PI3K/Akt signaling pathway

**DOI:** 10.3389/fonc.2023.1252999

**Published:** 2023-10-23

**Authors:** Dan Yu, Zhenlong Xiao, Zhefei Zou, Ling Lin, Jing Li, Jian Tan, Wei Chen

**Affiliations:** ^1^Department of Otorhinolaryngology, The Central Hospital of Wuhan, Tongji Medical College, Huazhong University of Science and Technology, Wuhan, China; ^2^Department of Otorhinolaryngology, The Central Hospital of Wuhan, Hubei University of Medicine, Shiyan, Hubei, China; ^3^Key Laboratory for Molecular Diagnosis of Hubei Province, The Central Hospital of Wuhan, Tongji Medical College, Huazhong University of Science and Technology, Wuhan, China; ^4^State Key Laboratory of Ultrasound in Medicine and Engineering, Chongqing Medical University, Chongqing, China

**Keywords:** IGF2BP2, HNSCC, tumorigenesis, miR-98-5p, PI3K/AKT

## Abstract

**Introduction:**

As a N6-methyladenosine reader protein, Insulin-like growth factor 2 mRNA-binding protein 2 (IGF2BP2) is a critical player in tumor progression and metastasis. However, its specific function in head and neck squamous carcinoma (HNSCC) has yet to be determined. The present study aimed to determine the role of IGF2BP2 in HNSCC.

**Methods:**

The expression of IGF2BP2 in HNSCC was analyzed using The Cancer Genome Atlas (TCGA) dataset and detected in HNSCC tissues and cells, respectively. Gain- and loss- of function methods were employed to study the effects of IGF2BP2 on HNSCC cell proliferation and tumorigenesis *in vitro and in vivo*. MicroRNAs (miRNAs) regulating IGF2BP2 were predicted using online tools and confirmed experimentally.

**Results:**

We showed augmented IGF2BP2 expression in HNSCC, which correlated with poor clinical outcomes. Functional studies showed that IGF2BP2 promoted HNSCC cell proliferation by facilitating cell cycle progression while inhibiting apoptosis. We further demonstrated that IGF2BP2 could enhance HNSCC cell tumorigenesis *in vivo*. Mechanistically, our data revealed that miR-98-5p could directly target *IGF2BP2*. The interplay between *IGF2BP2 and* miR-98-5p is essential to drive the progression of HNSCC via the phosphatidylinositol-4,5-bisphosphate 3-kinase (PI3K)-protein kinase B (Akt) pathway signaling pathway.

**Discussion:**

The current study revealed the oncogenic role of IGF2BP2 and provided insights into its potential mechanism in HNSCC tumorigenesis. Additionally, IGF2BP2 might represent a promising therapeutic target and serve as prognostic biomarker in patients with HNSCC.

## 1 Introduction

With an annual incidence of around 600,000 new cases and > 50% mortality, head and neck squamous cell carcinoma (HNSCC) ranks as the 6th most prevalent cancer globally (1, 2). Regardless of many advances in the treatment of HNSCC, such as surgical techniques, targeted therapies, and immunotherapy, the 5-year survival rate remains stubbornly low, at 40–50% (2, 3). The poor clinical outcomes of HNSCC can be attributed to various factors, including cancer stem cells that support cancer cell proliferation, tumor metastasis, recurrence, and chemoresistance (4). Consequently, conducting in-depth research on the molecular mechanisms and exploring the pathogenesis of HNSCC are important to develop effective treatments, with the ultimate goal of enhancing the overall survival rate of patients with HNSCC.

Insulin-like growth factor 2 mRNA-binding protein 2 (IGF2BP2) is a member of the evolutionarily conserved family of RNA-binding proteins known as IGF2BPs, which includes IGF2BP1 and IGF2BP3 (5, 6). Acting as a post-transcriptional regulator, IGF2BP2 is critical for the mRNA localization, translation, and stability (6, 7). Accumulating evidence suggests that IGF2BP2 is a major contributor to tumor initiation and development. It has been reported IGF2BP2 could facilitate colorectal cancer cell proliferation by inhibiting the degradation of replication factor A protein 1 (RFA1) (8). Another study showed that *IGF2BP2* maintains glioblastoma stem cell properties by mediating the silencing of a let-7 target gene (9). Furthermore, *in vivo* studies using *Igf2bp2* knockout mice showed that IGF2BP2 serves as an important regulator driving the progression of malignant tumors (10). Recent publications have identified IGF2BPs as a unique class of N6-methyladenosine (m6A) reader proteins that regulate and recognize m6A modification on mRNAs, which is important for their oncogenic functions (11) However, little is known about IGF2BP2’s function as an m6A reader protein in HNSCC.

MicroRNAs (miRNAs) are small non-coding RNAs (22–25 nucleotides long) that usually induce mRNA degradation or suppress translation after binding to the 3’-UTR of mRNAs. They are involved in virtually all biological activities, including development, metabolism, immune responses, proliferation, and differentiation (12–14). Accumulating evidence shows that dysregulated miRNA expression and function could result in tumorigenesis and cancer progression in humans (13–15). Specifically, recent studies shown that downregulation of miR-98-5p might contribute to the initiation and development of malignancies in various cancers (16, 17). Nonetheless, the specific functions of miR-98-5p in HNSCC remain elusive and require further investigation.

The present study aimed to determine the role of IGF2BP2 in HNSCC. The data showed increased expression of IGF2BP2 in HNSCC, which in turn promoted tumor cell proliferation through the phosphatidylinositol-4,5-bisphosphate 3-kinase (PI3K)-protein kinase B (Akt) pathway. We propose that this abnormal upregulation of IGF2BP2 is partially influenced by the posttranscriptional regulation mediated by the tumor suppressor miRNA-98-5p. Our analysis of clinical correlation and survival predictions further indicated that IGF2BP2 could be used as a reliable prognostic marker in HNSCC diagnosis. Overall, our findings highlight the significance of IGF2BP2 upregulation in the carcinogenesis of HNSCC and suggest its promising potential as a therapeutic target.

## 2 Materials and methods

### 2.1 HNSCC cell culture and tissue harvesting

Two HNSCC cell lines, FaDu and Detroit 562, were obtained from the Chinese Academy of sciences (Shanghai, China). SCC15, TSCCA, Cal-27, and human normal oral mucosal HOK cell lines were generously provided by Professor Kai Yang. They were cultured as follows: TSCCA cells were grown in Roswell Park Memorial Institute (RPMI)-1640 medium (Gibco, Grand Island, NY, USA), while other cells were maintained in Dulbecco’s modified Eagle’s medium (DMEM)(Gibco). All cell culture media were supplemented with 10% fetal bovine serum (FBS; PAN-Biotech, Aidenbach, Germany) and 1% penicillin-streptomycin. Furthermore, a total of 57 paraffin-embedded tissue specimens from patients with HNSCC were obtained from patients who underwent surgery in the Otolaryngology department of the First Affiliated Hospital (Jan. 2012- Dec. 2019, Chongqing, China). The clinical characteristics of all the patients are listed in **Table 1**. All patients completed informed consent forms before surgery. All experiments were approved by the First Affiliated Hospital of Chongqing Medical University.

**Table 1:** Relationship between *IGF2BP2* expression and the clinicopathological features of patients with HNSCC.

Characteristics	Total	*IGF2BP2* expression		χ^2^	P Value
		High	Low		
**Age(y)**				2.334	0.127
≥60	35	20	15		
<60	22	8	14		
**Sex**				1.054	0.305
male	56	27	29		
female	1	1	0		
**Alcohol**				0.001	0.971
yes	53	26	27		
no	4	2	2		
**Smoking**				8.489	0.004^*^
yes	44	17	27		
no	13	11	2		
**T classification**				6.484	0.013^*^
T1	4	1	3		
T2	8	3	5		
T3	27	18	9		
T4	18	6	12		
**Clinical stage**				18.038	< 0.001^*^
I	18	16	2		
II	8	1	7		
III	26	9	17		
IV	5	2	3		
**Tumor differentiation**				3.115	0.211
well	14	9	5		
moderate	30	15	15		
poor	13	4	9		

IGF2BP2, Insulin-like growth factor 2 mRNA-binding protein 2; HNSCC, Head and neck squamous carcinoma cells. The *P* Value was measured using a Chi-squared test. *P < 0.05. The bold values represent a p-value of less than 0.05, indicating statistical significance.

### 2.2 Immunohistochemistry staining

Tissue samples sections were deparaffinized in fresh xylene, followed by rehydration through graded alcohol. Next, the sections were subjected to heat-mediated antigen retrieval in sodium citrate buffer. After blocking endogenous peroxidases using goat serum, the sections were incubated with anti-IGF2BP2 primary antibodies overnight. After washing with phosphate-buffered saline (PBS), the sections were incubated with detection solution containing horseradish peroxidase-labeled streptavidin, followed by more PBS rinsing. Lastly, 3,3′-Diaminobenzidine (DAB) color development, hematoxylin staining, mounting with neutral gum, and imaging under a microscope were performed. Signal intensities of 0, 1, 2, and 3 was scored as no signal (0), weak (1), moderate (2), and strong (3); and staining distribution was recorded according to the range of positive cells: 0 (0–5%), 1 (5–25%), 2 (25–50%), 3 (50–75%), 4 (75– 100%). The cutoff was determined using the median value. All antibodies used in this study are listed in **Supplemental Table S2**.

### 2.3 Small interfering RNA and cell transfection

Three siRNAs targeting *IGF2BP2*, and a negative control (NC) siRNA, were acquired from GenePharma (Shanghai, China). In brief, 2 × 10^5^ HNSCC cells were seeded in each well of six-well plates and cultured until they reached 70% confluence. siRNAs were transfected into the cells using Lipofectamine iMAX Reagent (Invitrogen, Waltham, MA, USA) for 24 h in serum-free medium, followed by another 24 h culture using fresh complete cell culture medium. After 48 h or 72 h, knockdown of *IGF2BP2* was confirmed at the mRNA and protein levels using quantitative real-time reverse transcription PCR (qRT-PCR) and western blotting, respectively. The target sequences for *IGF2BP2 and* the NC can be found in **Supplemental Table S1**.

### 2.4 Lentivirus treatment to silence/overexpress IGF2BP2

Lentiviral vector GV344 encoding short hairpin RNAs (shRNA) against human *IGF2BP2* were constructed to silence human *IGF2BP2*. For *IGF2BP2* overexpression, lentiviral vector GV492 containing the full-length cDNA of human IGF2BP2 was generated. Both lentiviruses were produced by Genechem (Shanghai, China) and the target sequences are listed in **Supplement Table S1**. 5 × 10^4^ HNSCC cells were seeded in six-well plate and cultured until 30% confluence. Then, the cells were then infected with the lentiviral vectors at a multiplicity of infection (MOI) of 10 for 16 h, after which the viral medium was replaced with fresh medium. The cells were selected using puromycin (2 μg/ml)-containing culture medium for 1 week to establish stable *IGF2BP2* silenced or overexpressing HNSCC cells.

### 2.5 qRT-PCR

Total RNA was isolated using an E.Z.N.A.^®^ Total RNA Kit I (Omega Bio-tek, Winooski, VT, USA), followed by reverse transcription with the PrimeScript™ RT Reagent Kit with gDNA Eraser (Takara, Shiga, Japan). Next, the qPCR step of the qRT-PCR protocol was performed using a SYBR Premix Ex Tag™ Kit (Takara). Relative *IGF2BP2* and miR-98-5p expression levels were calculated using the 2^-ΔΔCt^ method (18), and were normalized to *GAPDH* and *U6* expression, respectively. The sequences of the primers used for qRT-PCR are listed in **Supplemental Table S1**.

### 2.6 Western blotting

Total proteins were extracted using a kit (KGP250, KeyGen, Jiangsu, China). Protein samples (30 μg) was separated by SDS-PAGE and transferred onto polyvinylidene fluoride (PVDF) membranes. After 2 h of blocking with 5% nonfat dry milk, we incubated the membranes with primary antibodies overnight at 4°C. Membranes were washed the next day, and then incubated with secondary antibodies for 1 h, followed by more washes. Lastly, the immunoreactive protein bands were visualized using an ECL kit (12043-D10, Advansta, San Jose, CA, USA) captured by a ChemiDoc Touch Imaging System (Bio-Rad, Hercules, CA, USA) and quantified using ImageJ (version v1.8.0). All antibodies used in this study are listed in **Supplemental Table S2**.

### 2.7 Cell growth assay

Cell viability was measured using a Cell Counting Kit-8 (CA1210, Solarbio, Beijing, China). Briefly, HNSCC cells were seeded at density of 2 × 10^3^ cells/well in 96-well plate. After 24 h, 10 μL of CCK-8 reagent was added to these cells and incubated for 1 h. Then, OD readings were taken at 450 nm.

### 2.8 Colony formation assay

Cells were cultured for 10–14 days starting at 1 × 10^4^ cells/well seeding density in six-well plates. Culture medium was refreshed every 2–3 days. At the end of the incubation, cells were stained with 0.1% crystal violet after fixing with 4% paraformaldehyde (PFA). Lastly, we measured colony numbers using inverted microscope.

### 2.9 Cell proliferation assay

After incubating cells with 5-Ethynyl-2’-deoxyuridine (EdU) working solution (C10310, RiBo, Guangzhou, China) for 4–6 h, we fixed the cells with 4% cold PFA for 30 min at room temperature. After permeabilizing the cells with Triton X-100 (0.5%, 15 min), the cells were treated with EdU reaction mixture for 30 min in the dark. Then, we stained the DNA using 4′,6-diamidino-2-phenylindole (DAPI) (C1005, Beyotime, Shanghai, China). Images were captured under a fluorescence microscope (Nikon, Tokyo, Japan).

### 2.10 Flow cytometry analysis

To measure cell apoptosis, HNSCC cells were cultured until 80% confluence. Then, the cells were stained with annexin V-fluorescein isothiocyanate (FITC)/propidium iodide (PI). To examine cell cycle status, we used a Cell Cycle Analysis Kit (C1052, Beyotime, Shanghai, China) in accordance with the manufacturer’s protocol. Lastly, these cells were analyzed using flow cytometry.

### 2.11 Xenograft model

Male BALB/cA nude mice (4 weeks old) were provided by Huafukang Biotechnology Co., (Beijing, China). To generate xenograft model, we subcutaneously injected 5 × 10^6^ si-*IGF2BP2* or si-NC treated FaDu cells (in 100 ul of PBS) into the left flank of the nude mice. At one week post injection, we recorded the tumor size using vernier calipers at 4-day intervals. The following formula was used to calculate the tumor volume: V (mm^3^) = 0.5 × length (longest diameter) × width (shortest diameter)^2^.

### 2.12 Luciferase reporter assay

A 3’-UTR segment of *IGF2BP2* and its mutant were inserted into luciferase expression reporter vector (Ribo Biotechnology Co., Guangzhou, China). To validate that miR-98-5p targets *IGF2BP2*, we co-transfected HNSCC cells with the luciferase vector and miR-98-5p mimic. After 48 h, luciferase activity was measured using a Dual-Luciferase® Reporter Assay System (E1910, Promega, Madison, WI, USA).

### 2.13 Statistical analysis

Data are expressed as means ± SD from at least three independent experiments. GraphPad Prism (version 7.0; GraphPad Inc., La Jolla, CA, USA) and SPSS 21.0 software (IBM Corp., Armonk, NY, USA) were used. Student’s t-test was performed to compare results between two groups; differences among multiple groups were evaluated using one-way analysis of variance (ANOVA). The chi-squared test was used to assess the correlation between *IGF2BP2* expression and clinicopathologic parameters. Survival rates were generated using the Kaplan–Meier method with log-rank statistics. P < 0.05 was used to define statistically significance; “NS” indicates no statistical significance.

## 3 Results

### 3.1 IGF2BP2 is upregulated in HNSCC

**Figure 1:**
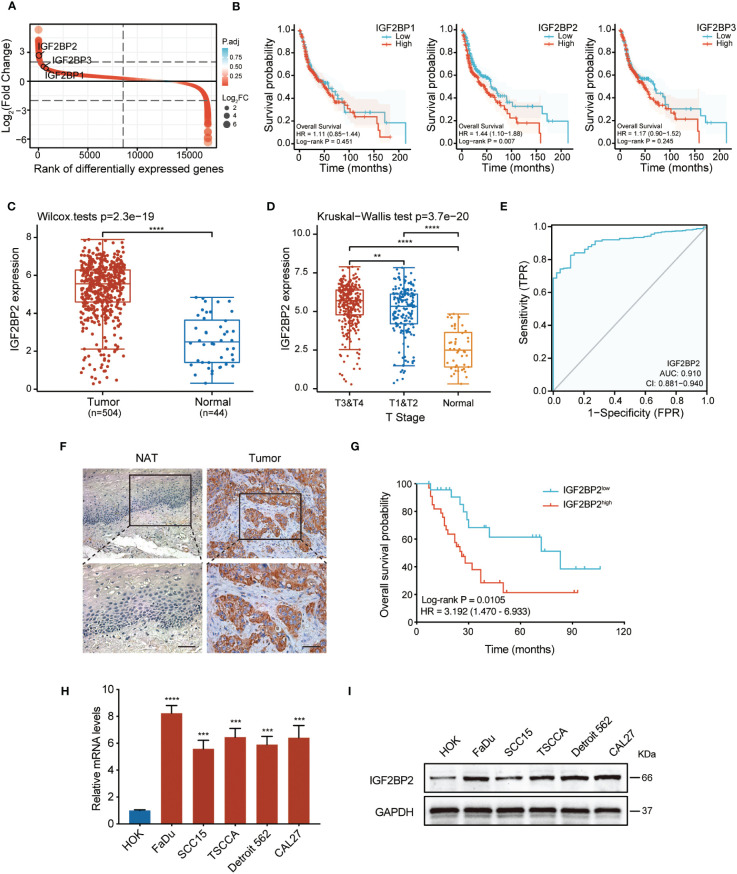
*IGF2BP2* is highly expressed in HNSCC and a potential prognostic marker. **(A)** A rank order plot showing differential expression of *IGF2BP* genes in HNSCC. **(B)** Survival analysis related to *IGF2BP1*, *IGF2BP2*, and *IGF2BP3* in patients with HNSCC based on TCGA data. The median was used as the cut-off value to categorize IGF2BP2 expression into two groups: ‘High’ (251 cases) and ‘Low’ (252 cases). **(C)** The expression analysis of *IGF2BP2* in 504 HNSCC and 44 normal tissues from the TCGA database. ****P < 0.0001. **(D)** The distribution of *IGF2BP2* expression in HNSCC tissues with different T stage tissues and normal tissues. **P < 0.01, ****P < 0.0001. **(E)** ROC curve analysis of the sensitivity and specificity of *IGF2BP2* in patients with HNSCC. AUC, area under the curve of the ROC curve analysis. **(F)** Representative images of IGF2BP2 expression in the paraffin-embedded HNSCC tissues and normal adjacent tissues (NATs). Scale bars: 200 μm. **(G)** Kaplan–Meier curves of OS in patients with HNSCC with low and high expression of *IGF2BP2*. The median *IGF2BP2* expression was applied as the cutoff value. qRT-qPCR **(H)** and western blotting **(I)** analysis of IGF2BP2 expression in HNSCC cell lines and normal oral mucosal HOK cells. GAPDH served as the internal control. All the data are presented in the form of mean ± SD from three independently performed experiments. ***P < 0.001, ****P < 0.0001.

Firstly, we analyzed the expression levels of the *IGF2BP* family in HNSCC tissues from the TCGA database. As shown in **Figure 1A**, the three genes were all upregulated in HNSCC, among which *IGF2BP2* showed the most prominent upregulation. Furthermore, we analyzed the correlation between the overall survival rate and *IGF2BP1*, *IGF2BP2*, and *IGF2BP3* expression in HNSCC in the TCGA dataset. Intriguingly, out of the three genes examined, only elevated expression of *IGF2BP2* correlated with decreased survival in patients with HNSCC (**Figure 1B**). Analyzing data from the TCGA dataset demonstrated that *IGF2BP2* was highly upregulated in HNSCC tumor tissues compared with that in healthy tissues (**Figure 1C**). Moreover, markedly increased expression levels of *IGF2BP2* from T3&T4 tissues and T1&T2 tissues were observed, in contrast to those in normal tissues (**Figure 1D**). In addition, the predictive performance of *IGF2BP2 in* HNSCC was measured by receiver operating characteristic (ROC) curve analysis, with an AUC value of 0.910 (**Figure 1E**). To assess its clinical significance, we conducted IHC analysis on 57 HNSCC cases with comprehensive clinicopathological information and follow-up data. The results showed that IGF2BP2 levels were higher in HNSCC samples compared with those in adjacent healthy tissues (**Figure 1F**). Importantly, increased IGF2BP2 levels correlated positively with poorer overall survival probability (**Figure 1G**). Moreover, analysis (shown in **Table 1**) demonstrated that *IGF2BP2* expression was associated with smoking, T classification, and clinical stage. Lastly, the qRT-PCR and western blotting results showed markedly higher IGF2BP2 mRNA and protein levels in all tested HNSCC cells (**Figures 1H, I**). Collectively, these data indicated that IGF2BP2 levels are enhanced in various HNSCC cell lines, which might contribute to their oncogenic phenotype.

### 3.2 IGF2BP2 promotes HNSCC cell proliferation

**Figure 2:**
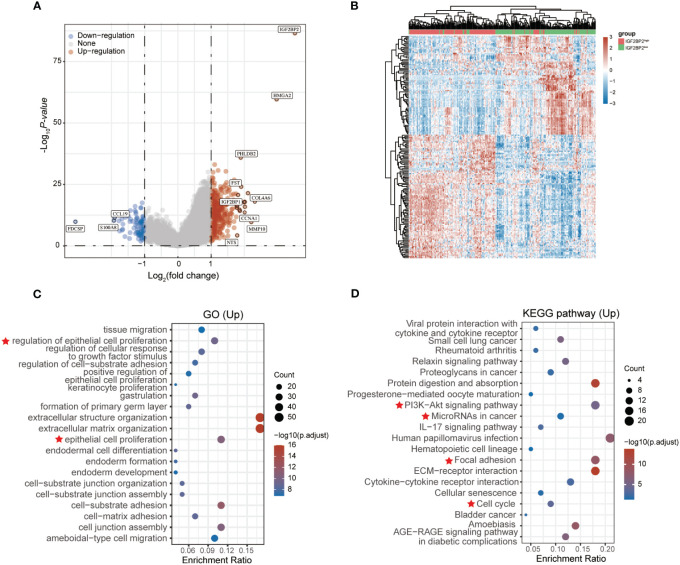
GO and KEGG analysis of the DEGs in HNSCC based on the TCGA dataset. Volcano plot **(A)** and heatmap **(B)** showing the differentially expressed genes (DEGs) between the high and low IGF2BP2 expression groups of patients with HNSCC. Red dots: upregulated genes; blue dots: downregulated genes; grey dots: not significant. Results of Gene ontology (GO) analysis **(C)** and KEGG pathway enrichment analysis **(D)** of DEGs. Red star represents the significantly enriched biological functions and signaling pathways in the IGF2BP2 high-expression group.

To assess the underlying function of IGF2BP2, data from the TCGA dataset was reorganized into different groups based on *IGF2BP2* expression, followed by differential expression analysis (|log2 FC| ≥ 2, adj. P < 0.05) (https://portal.gdc.com). As shown in **Figures 2A**, **B**, we identified 401 differentially expressed genes (321 upregulated and 80 downregulated). In addition, gene ontology (GO) analysis showed a substantial enrichment of genes related to cell proliferation in the IGF2BP2-high group (**Figure 2C**). Furthermore, Kyoto Encyclopedia of Genes and Genomes (KEGG) enrichment analysis indicated that most of them were enriched in pathways such as PI3K/Akt signaling, microRNA in cancer, and cell cycle (**Figure 2D**).

**Figure 3:**
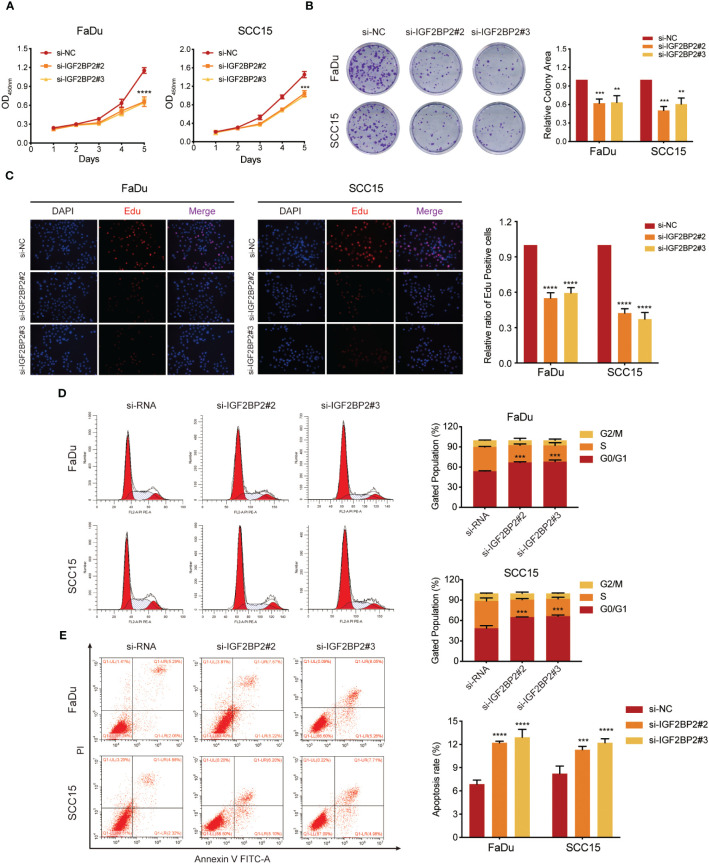
IGF2BP2 promotes cell proliferation of HNSCC cells *in vitro*. **(A)** CCK-8 assays of cell viability in FaDu and SCC15 cells. The viability of HNSCC cells was detected at the indicated times. **(B)** Representative images of colony formation assays of HNSCC cells. Right panel shows the statistical data of the relative colony area. **P < 0.01, ***P < 0.001. **(C)** Representative images of EdU incorporation assays in HNSCC cells. Right panel shows the quantification of EdU positive cells. ****P < 0.0001. **(D, E)** Flow cytometry assays of cell cycle distribution **(D)** and apoptosis **(E)** in FaDu and SCC15 cells. Lower right panel shows the quantification of cell proportions for each cell cycle phase and apoptotic cells. ***P < 0.001, ***P < 0.001. All data are presented as mean ± SD of three independent experiments.

To determine the impact of IGF2BP2 on HNSCC cell growth, we utilized an siRNA to downregulate *IGF2BP2* expression in FaDu and SCC15 cells. The knockdown efficiency was validated by western blotting (**Figures S2A, B**) As shown in **Figures 3A** and **B**, the CCK-8 and colony formation assays proved that *IGF2BP2* knockdown in HNSCC cells reduced their viability and growth. Consistently, silencing *IGF2BP2* notably suppressed the proliferation in FaDu and SCC15 cells, as shown by Edu analysis (**Figure 3C**). Flow cytometry assays revealed that *IGF2BP2* silencing could induce G0/G1 phase cell cycle arrest and promoted apoptosis (**Figures 3D, E**). Overall, these data suggested that IGF2BP2 promotes HNSCC cell growth by facilitating cell cycle progression and suppressing apoptosis.

### 3.3 IGF2BP2 enhances HNSCC cell tumorigenesis *in vivo*

**Figure 4:**
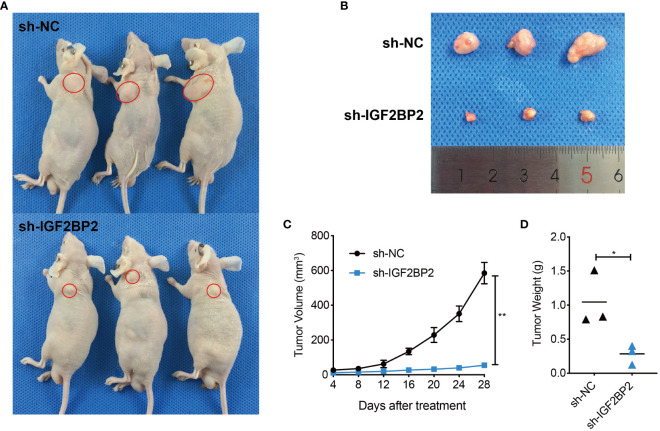
IGF2BP2 enhances HNSCC cell tumorigenesis *in vivo.* **(A, B)** Representative images of subcutaneous xenograft tumors of sh-IGF2BP2 and sh-NC groups in nude mice (n = 3 per group). **(C)** The growth curve of the xenograft tumors originating from sh-IGF2BP2 and sh-NC groups. **(D)** The weight of xenograft tumors for the sh-IGF2BP2 and sh-NC groups. *P < 0.05, **P < 0.01. All the data are presented in the form of mean ± SD from three independently performed experiments.

To further validate the function of IGF2BP2 in promoting oncogenesis of HNSCC, a xenograft model was generated by subcutaneous injection of IGF2BP2-silenced FaDu cells into mice. The silencing efficiency of IGF2BP2 was verified by western blot (**Figure S2C**). Interestingly, the sh-IGF2BP2 group exhibited smaller sized tumors compared with those of the sh-NC group (**Figures 4A, B**). In addition, the sh-IGF2BP2 group showed a significantly slower tumor growth rate than the sh-NC group (P < 0.05) (**Figure 4C**). Meanwhile, the tumor weight from the sh-IGF2BP2 group was substantially lower than that from the sh-NC group (**Figure 4D**). Altogether, these results demonstrated that silencing *IGF2BP2* suppressed HNSCC cell growth in the animal model.

### 3.4 miR-98-5p directly targets *IGF2BP2*

**Figure 5:**
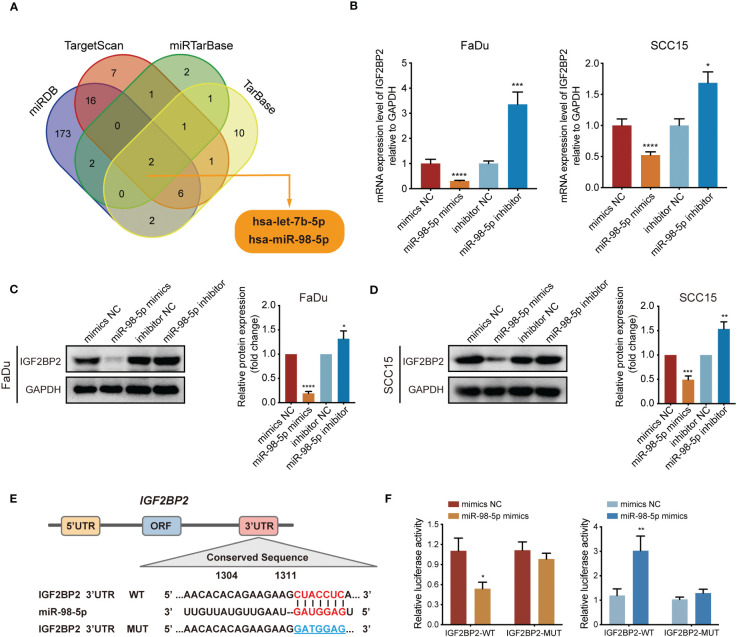
*IGF2BP2* is a direct target of miR-98-5p. **(A)** Venn diagram showing the overlap microRNAs from four microRNA prediction algorithms. **(B)** qRT-PCR analysis of *IGF2BP2* mRNA expression levels in FaDu and SCC15 cells treated with miRNA-98-5p mimics or inhibitors. *P < 0.05, ***P < 0.001, ****P < 0.0001. **(C, D)** Western blotting analysis of IGF2BP2 protein levels in FaDu and SCC15 cells treated with miRNA-98-5p mimics or inhibitors. Right panel shows the statistical analysis of the western blot. **(E)** An illustration of the predicted binding site for miR-98-5p in the 3′-UTR of *IGF2BP2*. **(F)** Luciferase activity assays showing the direct binding efficiency of miR-98-5p and its putative *IGF2BP2* 3′-UTR target. Data are presented as the mean ± SD of three independent experiments. *P < 0.05, **P < 0.01.

MicroRNAs (miRNAs) are essential players in cancer development and progression. According to the results of KEGG analysis in **Figure 2D**, a question arose as to whether the dysregulated miRNA levels would affect the expression of *IGF2BP2*. To address this question, we used TargetScan, miRTarBase, miRDB, and TarBase databases to screen microRNAs that can target *IGF2BP2*, and two candidate miRNAs were identified (**Figure 5A**). Upon analyzing the TCGA database for HNSCC, we discovered a significant downregulation of miR-98-5p in *IGF2BP2*^high^ HNSCC tissues, in comparison with that in the *IGF2BP2*^low^ HNSCC group (**Figure S1A**). However, there was no significant differential expression of let-7b-5p between *IGF2BP2*^high^ and *IGF2BP2*^low^ HNSCC tissues (**Figure S1B**). These findings indicated a potential negative regulatory association between *IGF2BP2* and miR-98-5p. Consequently, we studied the functional roles of miR-98-5p by transfecting the miR-98-5p mimic into FaDu cells and the miR-98-5p inhibitor into SCC15 cells. Notably, miR-98-5p exhibited robust suppression on IGF2BP2 mRNA and protein levels (**Figures 5B–D**). To confirm if miR-98-5p can directly target *IGF2BP2*, luciferase experiments were performed using the *IGF2BP2* 3′-UTR linked to the luciferase coding region (**Figure 5E**). When comparing the miR-98-5p mimic group with the control group, a substantial decrease in luciferase activity was observed (**Figure 5F**). However, luciferase activity remained unchanged when miR-98-5p mimics were transfected into the group with mutated *IGF2BP2* 3′-UTR. Overall, our results provide support for the direct binding of miR-98-5p to the 3′-UTR of *IGF2BP2*, thus downregulating IGF2BP2 translation.

### 3.5 miR-98-5p suppresses tumor growth by downregulating *IGF2BP2*

**Figure 6:**
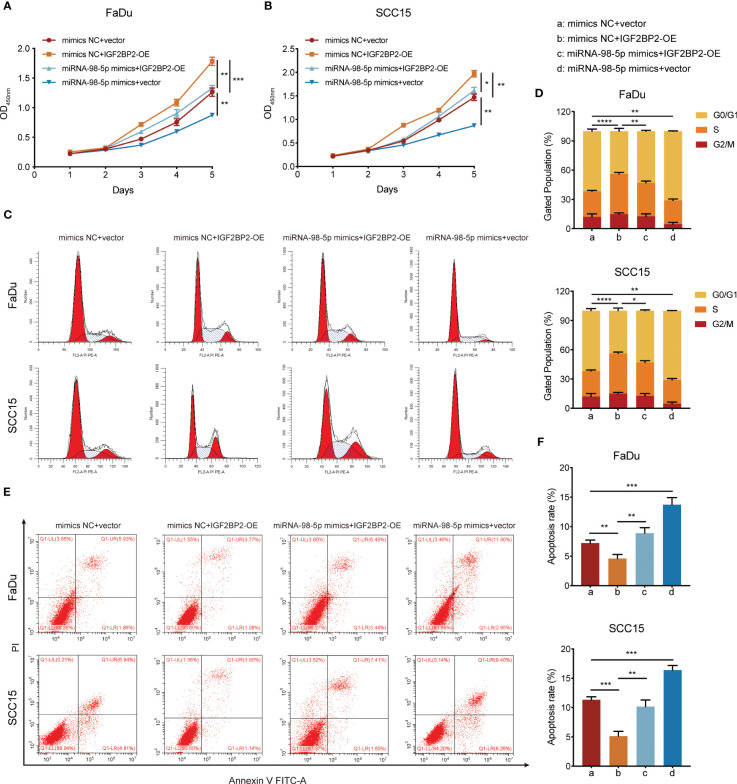
miR-98-5p negatively mediates the oncogenic activity of IGF2BP2. **(A, B)** CCK-8 assays determining the effect of miR-98-5p transfection after IGF2BP2 overexpression on HNSCC cell proliferation. The viability of HNSCC cells was detected at the indicated times. *P < 0.05, **P < 0.01, ***P < 0.001. **(C, D)** Cell cycle determined by flow cytometry following *IGF2BP2* overexpression or transfection of miR-98-5p mimics. Right panel shows the quantification of cell proportions for each cell cycle phase. **P < 0.01, ***P < 0.001. **(E, F)** Cell apoptosis determined by flow cytometry following *IGF2BP2* overexpression or transfection of miR-98-5p mimics. Right panel shows the quantification of apoptotic cells. *P < 0.05, **P < 0.01, ***P < 0.001. All data are presented as the mean ± SD of three independent experiments.

To investigate whether miR-98-5p suppresses tumor cell growth facilitated by IGF2BP2, we overexpressed *IGF2BP2*. Subsequently, these cells were transfected with miR-98-5p mimics or the negative control vector. The overexpression efficiency of *IGF2BP2* was validated in our previous study (19). The CCK-8 analysis demonstrated that upregulation of *IGF2BP2* significantly boosted the proliferation capacity of FaDu and SCC15 HNSCC cell lines. However, this effect was partially counteracted by re-introducing miR-98-5p (**Figures 6A, B**). In addition, flow cytometry assays showed that *IGF2BP2* overexpression decreased G0/G1 phase proportion and inhibited apoptosis. However, re-expression of miR-98-5p partially reversed these process in HNSCC cells (**Figures 6C–F**). These findings collectively supported the view that miR-98-5p negatively regulates HNSCC tumor cell proliferation.

### 3.6 IGF2BP2 activates PI3K/Akt signaling during HNSCC progression

**Figure 7:**
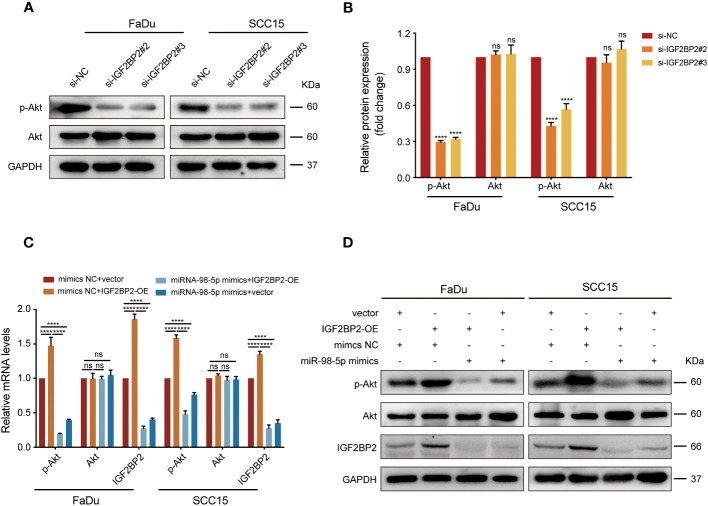
IGF2BP2/miR-98-5p contributes to progression of HNSCC via the PI3K/Akt signaling pathway. **(A, B)** FaDu and SCC15 cells were transfected with si-RNAs or si-NC. The levels of p-Akt and Akt were assessed using western blot analysis (left) and analyzed quantitatively (right). ****P < 0.0001. **(C, D)** *IGF2BP2*-overexpressing FaDu cells or corresponding control cells underwent transfection with miR-98-5p mimics or mimics NC. *AKT* and *IGF2BP2* expression identified by qRT-qPCR **(C)** and p-Akt, Akt, and IGF2BP2 levels detected by western blot **(D)**. ****P < 0.0001. GAPDH served as an internal or negative control. Data are presented as the mean ± SD of three independent experiments. ****P < 0.0001. ns, not statistically significant.

As shown from the results of the KEGG analysis, PI3K/Akt signaling was markedly enriched in *IGF2BP2*-upregulated cases (**Figure 2D**), which might be the key contributor to the progression of HNSCC. To validate this hypothesis, we performed western blotting and discovered that phosphorylation of Akt (p-Akt) was suppressed after *IGF2BP2* knockdown (**Figures 7A, B**). By contrast, *IGF2BP2* overexpression led to a marked increase in p-Akt levels in FaDu and SCC15 cells, which was attenuated by transfection with miR-98-5p mimics (**Figures 7C, D**). Collectively, these data show that IGF2BP2 regulates HNSCC cell growth by regulating the PI3K-Akt signaling pathway.

## 4 Discussion

It was suggested that IGF2BP2 might contribute to the pathogenesis and development of various tumors (20, 21). Our previous study revealed that IGF2BP2 could contribute to lymphatic metastasis of HNSCC (19). However, further exploration of IGF2BP2’s functions in HNSCC are required. In the current study, we detected increased IGF2BP2 levels in HNSCC, which correlated with unfavorable clinical outcomes. In addition, we discovered that IGF2BP2 could enhance tumorigenesis of HNSCC via activating PI3K/Akt signaling. Additionally, we demonstrated a direct interaction between *IGF2BP2* and miR-98-5p, which might regulate the oncogenic effects of IGF2BP2.

IGF2BP2, an RNA binding protein (RBP), was recognized recently as a member of unique class of m6A readers that play oncogenic roles in cancers (11). Recent studies have shown increased expression of IGF2BP2 in cancer cells, and its expression levels have been shown to be associated with unfavorable prognosis (21, 22). In this study, we identified 401 differentially expressed genes (e.g., HMGA2, PHLDB2, FST) associated with IGF2BP2 based on the TCGA dataset. Remarkably, these genes align with the mRNA targets of IGF2BP2 previously identified by Huang et al. (23). However, these studies mainly relied on the data mined from public database like the TCGA, and lacked validation of clinical samples and functional studies. Here, we validated that IGF2BP2 was markedly upregulated in samples from 57 patients with HNSCC and in 5 tested HNSCC cell lines. In addition, higher IGF2BP2 expression correlated significantly with low overall survival probability, suggesting that IGF2BP2 is a potential prognostic marker for HNSCC studies. Accumulating evidence links IGF2BP2 overexpression with cancer initiation and progression. Huang et al. (23) previously reported that IGF2BP2 downregulation exerts remarkably suppresses cells proliferation and invasion in non-small cell lung cancer (NSCLC). Furthermore, Mu et al. (24) reported that IGF2BP2 activates the PI3K/AKT signaling pathway, thereby promoting glioblastoma (GBM) cell growth, migration, and invasion. The present study provides further evidence that IGF2BP2 plays crucial roles in promoting HNSCC cell growth by facilitating cell cycle progression, as well as inhibiting apoptosis. Furthermore, *in vitro* experiments revealed that IGF2BP2 enhances tumorigenesis of HNSCC cells. Overall, our findings suggested that IGF2BP2 is an important player in HNSCC tumorigenesis.

Dysregulated microRNAs are associated with various types of cancer, including HNSCC (25). Through KEGG enrichment analysis, we observed a significant association between elevated *IGF2BP2* expression and microRNAs in cancer. Typically, miRNAs promote target mRNA degradation, which inhibits translation (13). Through comprehensive bioinformatic analysis and experimental validation, we confirmed that miR-98-5p can target *IGF2BP2* by direct binding to its 3′- UTR, which showed notable downregulation in HNSCC tissues after overexpressing miR-98-5p.

Results from further experiments demonstrated that miR-98-5p re-expression could partially suppress the oncogenic activity of IGF2BP2, further validating *IGF2BP2* as a direct target of miR-98-5p. Similarly, Wang et al. (17) reported that miR-98-5p triggered mesenchymal stem cell apoptosis by targeting IGF2BP1 via the PI3K/Akt pathway. Fu et al. (16) revealed that miR-98-5p downregulation caused suppression of mitogen-activated protein kinase (MAPK)/extracellular regulated kinase (ERK) signaling, and subsequently triggered pancreatic ductal adenocarcinoma (PDAC) proliferation and metastasis. Overall, these findings provide strong evidence that miR-98-5p could negatively regulate IGF2BP2 oncogenic activity in HNSCC.

Bioinformatic analysis was conducted to further dissect the molecular mechanism through which IGF2BP2 affect the progression of HNSCC. Interestingly, we detected a significant correlation between increased IGF2BP2 levels and PI3K/Akt signaling activation. Moreover, miR-98-5p re-expression partially attenuated Akt phosphorylation in HNSCC. Consistently, Xu et al. (19) demonstrated that IGF2BP2 promotes pancreatic cancer cell growth via the PI3K/Akt signaling cascade. Liu et al. (26) indicated that IGF2BP2 regulates vasculogenic mimicry formation via promoting signaling through PI3K/AKT/mTOR axis in colorectal cancer (CRC), further supporting the importance of the IGF2BP2/miR-98-5p axis in promoting HNSCC progression by modulating PI3K/AKT signaling. However, the regulatory mechanism underlying the activation of the PI3K/Akt pathway by IGF2BP2 is not yet fully understood. Mu et al. (24) reported that IMP2 regulates the activity of IGF2, subsequently activating the PI3K/Akt signaling pathway and promoting GBM malignancy. Shao et al. (27) revealed that miR-24-3p is involved in adipogenesis and lipid accumulation by targeting the IGF2/PI3K-AKT-mTOR axis. These studies suggest that IGF2 or IGF1R could be potential targets through which IGF2BP2 activates the PI3K/Akt signaling pathway. However, whether IGF2BP2 activates the PI3K/Akt signaling pathway through IGF2 or IGF1R requires further experimental verification.

Overall, the current study highlights the markedly upregulated IGF2BP2 levels in HNSCC, which activates PI3K-Akt signaling and plays a pivotal role in promoting tumor cell proliferation. This dysfunctional IGF2BP2 upregulation is attributed, at least partially, to posttranscriptional regulation by miRNA-98-5p. Furthermore, our data also established IGF2BP2 as a novel promising prognostic biomarker in patients with HNSCC. The results of the present study underscore the importance of IGF2BP2 in the carcinogenesis of HNSCC and suggest its potential as a therapeutic target.

## Data availability statement

The raw data supporting the conclusions of this article will be made available by the authors, without undue reservation.

## Ethics statement

The studies involving humans were approved by the Ethics Committee of the First Affiliated Hospital of Chongqing Medical University. The studies were conducted in accordance with the local legislation and institutional requirements. The participants provided their written informed consent to participate in this study. The animal study was approved by the Ethics Committee on Animal Research of the First Affiliated Hospital of Chongqing Medical University. The study was conducted in accordance with the local legislation and institutional requirements.

## Author contributions

WC and DY conceived and designed the study. DY performed the experiment with support from ZZ and LL. DY wrote the manuscript. WC supervised the experiments and approved it. JL, JT, and ZX aided in collecting patients’ specimens. All authors contributed to the article and approved the submitted version.

## Supplementary material

Supplementary Figure 1 The expression levels of miR-98-5p and let-7b-5p in IGF2BP2^high^ and IGF2BP2^low^ HNSCC tissues.

Supplementary Figure 2 The knockdown efficiency of IGF2BP2 in HNSCC cells was validated using western blot.
