# Supplementary material for: IGF2BP2 promotes head and neck squamous carcinoma cell proliferation and growth via the miR-98-5p/PI3K/Akt signaling pathway
Source: Front Oncol. 2023 Oct 23;13:1252999. doi: 10.3389/fonc.2023.1252999 (PMC10627011; doi:10.3389/fonc.2023.1252999)
Supplement: Supplementary file 1 [file Table_1.docx]

**Table S1. Primers used in the experiments.**

| **Gene name** | **Primer sequence** | | **Application** |
| --- | --- | --- | --- |
| IGF2BP2 | F | 5′-AGTGGAATTGCATGGGAAAATCA-3′ | qRT-PCR |
|  | R | 5′-CAACGGCGGTTTCTGTGTC-3′ |  |
| GAPDH | F  R | 5’-CAGCGACACCCACTCCTC-3′  5’-TGAGGTCCACCACCCTGT-3′ | qRT-PCR |
| miR-98-5p | mimic | 5′-UGAGGUAGUAAGUUGUAUUGUU--3′ | qRT-PCR |
|  | inhibitor  NC | 5′-CUAUACAACUUACUACUUUCCC-3′  5′-ACGCGTCGACTTAGCGTGGATTT-3′ |  |
| si-NC | sense  anti-sense | 5’-UUCUCCGAACGUGUCACGUTT-3′  5’-ACGUGACACGUUCGGAGAATT-3′ | si-RNA |
| si-IGF2BP2#1 | sense  anti-sense | 5'-GCGAAAGGAUGGUCAUCAUTT-3'  5'-AUGAUGACCAUCCUUUCGCTT-3' | si-RNA |
| si-IGF2BP2#2 | sense  anti-sense | 5'-GCUGUUAACCAACAAGCCATT-3'  5'-UGGCUUGUUGGUUAACAGCTT-3' | si-RNA |
| si-IGF2BP2#3 | sense  anti-sense | 5'-ACAGGACUGUCCGUGCUAUTT-3'  5'-AUAGCACGGACAGUCCUGUTT-3' | si-RNA |
| sh-NC |  | 5′-UUCUCCGAACGUGUCACGU-3' | sh-RNA |
| sh-IGF2BP2 |  | 5'-GCGAAAGGAUGGUCAUCAUTT-3’ | sh-RNA |

Abbreviations: F: Forward; R: Reverse; qRT-PCR: quantitative real-time PCR; si-RNA: Small interfering-RNA; sh-RNA: Short hairpin-RNA
