# Supplementary material for: IGF2BP2 promotes head and neck squamous carcinoma cell proliferation and growth via the miR-98-5p/PI3K/Akt signaling pathway
Source: Front Oncol. 2023 Oct 23;13:1252999. doi: 10.3389/fonc.2023.1252999 (PMC10627011; doi:10.3389/fonc.2023.1252999)
Supplement: Supplementary file 2 [file Table_2.docx]

**Table S2. Antibodies used in the experiments.**

| **Antibody** | **Source** | **No. of Catalogue** | **Dilution** | | |
| --- | --- | --- | --- | --- | --- |
|  |  |  | **WB** | **IHC** |  |
| IGF2BP2 | Abcam, USA | ab124930 | 1:2000 | 1:100 |  |
| p-Akt | Cell Signaling Technology, USA | 23430 | 1:1000 |  |  |
| Akt | Cell Signaling Technology, USA | 2920 | 1:1000 |  |  |
| GAPDH | Proteintech, China | 10494-1-AP | 1:3000 |  |  |

Abbreviations: WB: Western blot; IHC: Immunohistochemistry; IF: Immunofluorescence
