# Supplementary figures and images for: IGF2BP2 promotes head and neck squamous carcinoma cell proliferation and growth via the miR-98-5p/PI3K/Akt signaling pathway
Source: Front Oncol. 2023 Oct 23;13:1252999. doi: 10.3389/fonc.2023.1252999 (PMC10627011; doi:10.3389/fonc.2023.1252999)

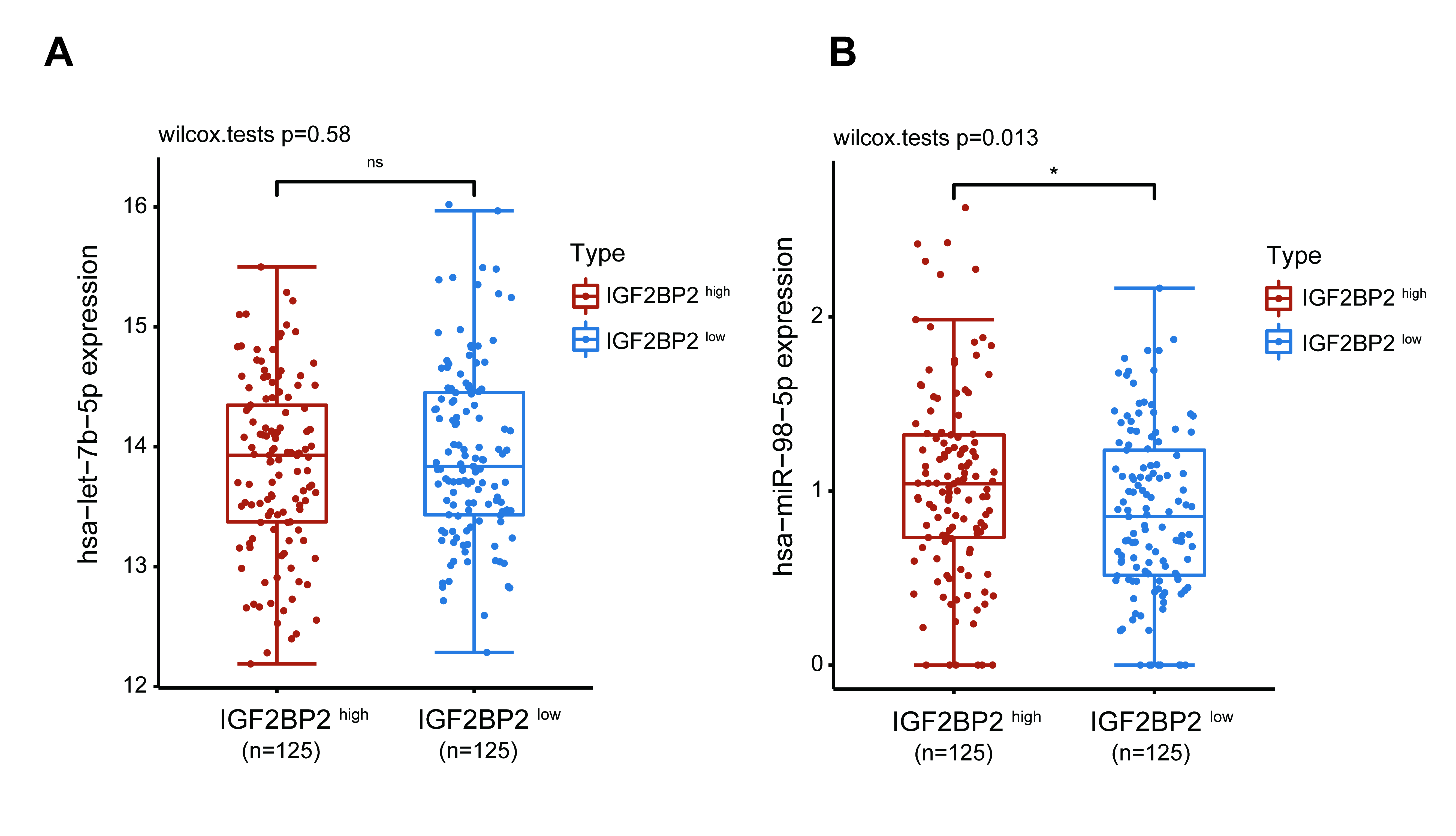

Supplement: Supplementary Figure 1 — The expression levels of miR-98-5p and let-7b-5p in IGF2BP2high and IGF2BP2low HNSCC tissues. [file Image_1.tif]

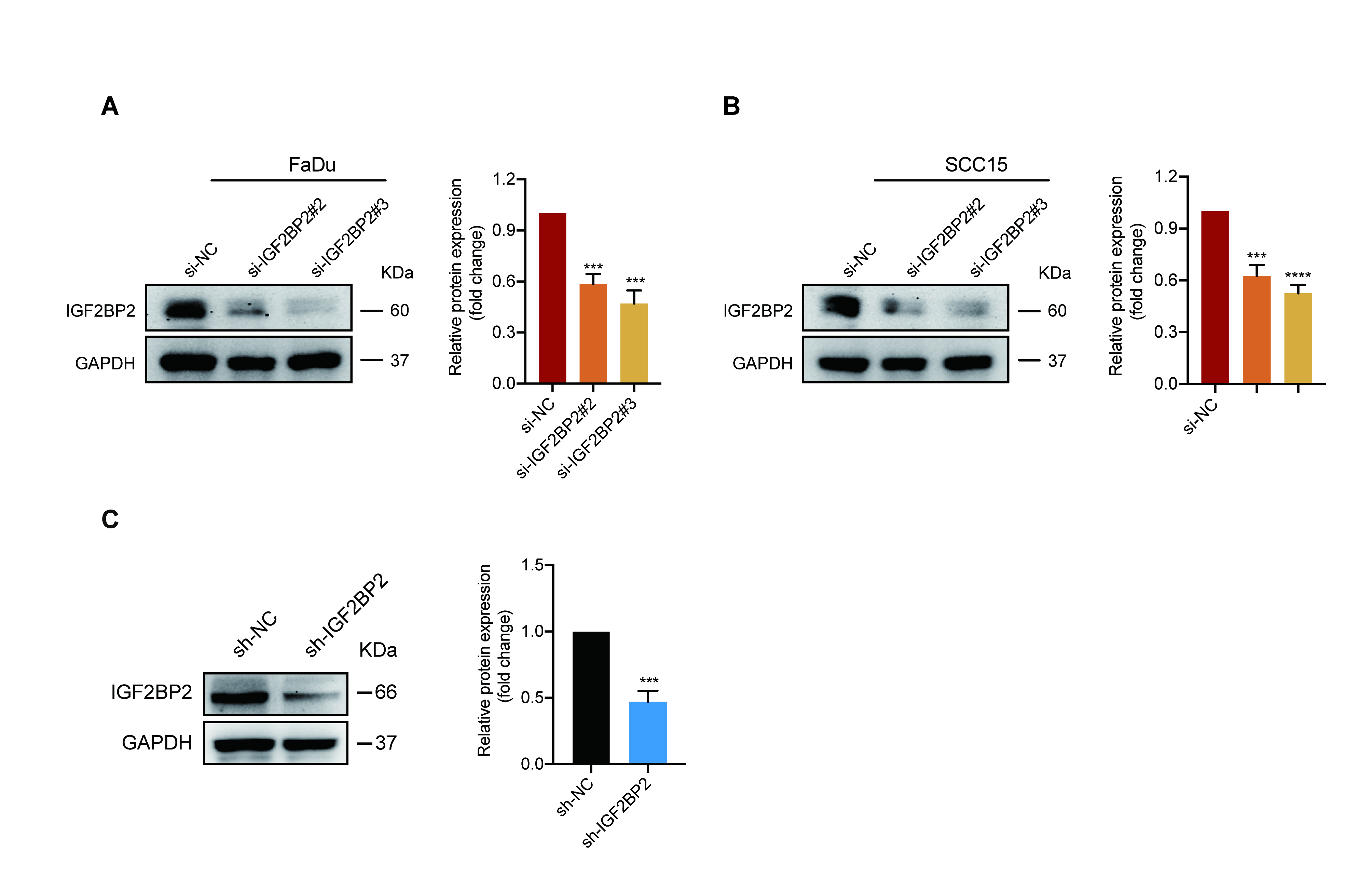

Supplement: Supplementary Figure 2 — The knockdown efficiency of IGF2BP2 in HNSCC cells was validated using western blot. [file Image_2.tif]
